# Increasing Interest in Child and Adolescent Psychiatry During Medical School: Launching a Summer Immersion Experience for Medical Students

**DOI:** 10.1007/s40596-021-01573-1

**Published:** 2021-12-23

**Authors:** Desirée N. Shapiro

**Affiliations:** University of California, San Diego, La Jolla, CA USA

There is a global shortage of child and adolescent psychiatrists (CAPs) with an insufficient supply to meet a growing demand. The World Health Organization estimates that there are less than 0.1 CAPs per 100,000 youth in all income levels globally, except in high-income regions that still only have 1.19 CAPs per 100,000 youth [1]. The American Academy of Child and Adolescent Psychiatry confirms the concerning shortage of CAPs in states across the nation [2]. There are approximately 9787 actively practicing CAPs in the USA with an estimated 9.75 CAPs per 100,000 youth with a need for at least 47 per 100,000 youth [3–6]. Despite a recent reassuring workforce projection, more action is needed and there is concern for underestimation of these projections [5–7]. In addition, there is a critical underrepresentation of certain racial and ethnic minoritized groups and women in medicine [8]. From the data available comparing the demographics and identities of the psychiatry workforce with the US population, gaps and opportunities are evident with female and historically marginalized groups being underrepresented [9]. The US population is increasingly becoming more multiracial and ethnically and racially diverse with significant diversity in our nation’s younger population [10–12]. It is estimated that by 2060, the number of children identifying as two or more races will double [13].

Equity, inclusion, and diversity must be central in education, care, and recruitment of the workforce pipeline. A diverse workforce is better able to respond to the needs of populations from a variety of backgrounds in clinical care, advocacy, teaching, and research; in addition, diversity drives excellence and improves outcomes, performance, and creativity [14]. It is imperative to consider ways to grow the CAP workforce and emphasize the urgency of representation to reflect communities in need of care, increase access, and improve patient experience and outcomes.

Efforts to grow a more inclusive workforce must begin early in the many pathways to CAP. Exposure to CAP during undergraduate medical education is one strategy to increase interest; however, this exposure varies widely and because of the demand for CAPs in clinical coverage, it is not always possible to learn pediatric mental health concepts from CAPs [15]. Early exposure and recruitment as well as positive role models have been found to be important when exploring factors that impact career decision-making [16–18]. Early introduction to CAP with invested teachers, mentors, and sponsors has the potential to increase awareness about the specialty. Reviews of enrichment programs in psychiatry conclude that these experiences may be promising strategies to increase exposure and interest in psychiatry careers [18, 19]. Notably, one program sponsored by the Klingenstein Third Generation Foundation offering mentorship, clinical experiences, and research exposure was found to increase knowledge and understanding of CAP as well as provide positive experiences with mentorship [17]. Psychiatry pipeline programs do exist, including fellowships through the American Psychiatric Association for medical trainees; however, there is limited outcome data providing guidance on how to successfully grow a more diverse workforce within CAP in one’s region [20].

This article describes the implementation and evaluation of a summer immersion program in CAP that aimed to recruit a diverse group of medical students and integrate cultural empathy and sensitivity throughout the program. Participation in this program was hypothesized to increase interest in the field of CAP and pediatric mental health.

## Program Description

The primary aim of the program was to increase exposure to CAP and pediatric mental health topics among medical students. Secondary aims included 1) recruiting a diverse group of medical students considering culture, ethnicity, race, gender, sexual orientation, language, religion, spirituality, background, personal identity, class, ability status, and experience and 2) increasing consideration of CAP as a career.

The program consisted of multiple virtual components including educational sessions on core CAP topics, a community engagement presentation for local adolescents, completion of a pediatric mental health project, individual mentorship, and encouragement of cohort cohesion and connection. Educational sessions were led by pediatric mental health experts each day of the week. Students also researched and delivered a presentation to their cohort on a mental health topic of their choice. Topics covered in the program are included in Table 1.

**Table 1:** Topics covered in the program

Introduction to child and adolescent psychiatry
Interviewing in CAP
Psychotherapy
Development
Trauma
Psychopharmacology
Research methods
Cultural humility in CAP: working with children and families and embracing our differences
Substance use and comorbidities
Grief
Anxiety
ADHD
Depression and mood disorders
Violence and youth
Cultural psychiatry
Cultural considerations and mental health: Middle Eastern communities*
Cultural considerations and mental health: LGBTQIA + communities*
Cultural considerations and mental health: Asian American Pacific Islander communities*
Cultural considerations and mental health: Latinx communities*
Disruptive, impulse, control, and conduct disorders
Structural determinants of health
Youth suicide
Sleep
LGBTQIA + racial minority youth mental health
Psychosis
Obsessive compulsive disorder
Disruptive, impulse, control, and conduct disorders
Eating and feeding concerns
School mental health
Social determinants of health
Self-compassion

*Delivered by a community-based outreach and education program

The community engagement project involved partnering with a group of adolescents representing six local high schools and collaboratively delivering a mental health workshop that honored youth perspective and voice throughout the planning and implementation of the workshop. Students also completed a summer project related to child, adolescent, or family mental health. Students engaged a variety of projects that ranged from scientific writings to community action projects. Students met with the program leadership regularly throughout the summer to discuss questions, reflections, individual learning goals, projects, as well as well-being, navigating the medical journey, and career options. Group discussion sessions occurred once or twice a week to reflect on what was learned. Students created a safe and nurturing space to dialogue about the relevance of mental health in their professional and personal lives. Cultural understanding, humility, and sensitivity were core themes of the discussion sessions. There was also opportunity to discuss the journey of medical school; fourth year students generously shared perspectives and experiences with more junior medical students. In addition, trainees benefitted from dialoguing about reasons why they may not want to pursue CAP, including lack of exposure, multiple forms of stigma, lengthy training, limited financial compensation, emotionally challenging work, and limited access to mentors/sponsors.

A WhatsApp group was used to communicate throughout the week about program content in addition to social events, happy hours, and movie nights. Some students attended additional events that were offered including a summer student led book club, Psychiatry and CAP Departmental Grand Rounds, community mental health meetings, county mental health trainings, Schwartz Rounds, and campus-wide events related to health equity and anti-racism.

Students applied in the winter of 2020 and were invited into the program in the spring of 2020. Students began exploring areas of interest in the spring of 2020 to start the summer with a plan for their pediatric mental health–focused projects. During the active phase of the 2-month program, students generally attended sessions, discussions, or meetings in the first half of the day and were expected to work on their individual projects, complete readings, meet for individual check-ins, or join an optional discussion in the afternoons. Student time on project development and implementations varied depending on the type of project and role. Faculty time during the spring and summer was considerable in planning, teaching, coordinating, and facilitating the program.

Students from the MD-only degree program received a financial stipend for participating. No students received academic credit for this experience; however, many students engage in a scholarly or research program in between the summer of year 1 and year 2 of medical school.

All applicants were asked to submit a statement of interest and a statement of equity, diversity, and inclusion. During recruitment, applicants with diverse backgrounds, identities, and life experiences were strongly encouraged and invited to apply. The 9 participants self-identified in the following ways: Asian/Asian American (*n* = 3), Black/African American (*n* = 1), Hispanic/Latinx (*n* = 1), White/Caucasian (*n* = 3), and declined to state (*n* = 1). Six participants were between 22 and 25 years of age, and three were between 26 and 29 years of age; six identified as female and three identified as male. The participants included six rising second year medical students, two rising fourth year students, and one student entering into a PhD program. The program was made possible by a grant from UnitedHealth Group.

## Evaluation Data

External evaluators from a local Center for Research and Evaluation reviewed the 2020 summer program using a post-program survey and 90-min focus group. Eight of the nine program participants completed the survey and eight out of nine completed the focus group. Completing the survey or focus group was optional and these were scheduled during a busy end of summer and beginning of the academic year period.

The feedback from the post-program survey and focus group was overwhelmingly positive, with all eight respondents reporting they were “very satisfied” with the program and “more likely” to pursue a CAP career because of their participation in the program. Half responded that they were “somewhat likely” to pursue CAP and the other half reported they were “very likely” to pursue CAP as a career. The youth outreach presentation and individual mentoring sessions were rated as the most useful program components. Themes from the focus groups included 1) an appreciation of the community of the cohort, 2) the opportunity to engage with and identify with diverse CAPs from across the country, 3) the faculty leadership and method of running the experience including commitment, availability, and encouragement, 4) perception shifts including dispelling popular and preconceived notions about CAP, and 5) the focus on cultural sensitivity and gaining comfort in talking about CAP concepts.

Feedback from participants included helpful suggestions including giving more lead time to develop summer projects, offering more time to process and debrief after sessions, including an early session on detailed career paths and options to become a CAP, and creating longitudinal opportunities to work with youth partners. Students also gave feedback on the talks and suggested additional topics to be added to the didactic series. Below are quotes from participants post-program.

This program offered a unique experience for medical students to dive into a field of medicine that is not emphasized during medical school. It was incredible to hear from experts in the field share their thoughts on child and adolescent psychiatry (CAP), the nuances of the field, their experiences with CAP. It opened our eyes to this extraordinary field that is challenging yet rewarding in every sense. The program also provided a much needed space for students to process medical school experiences such as being on wards in addition to processing the current and long-lasting injustices of the United States climate. In having a comfortable, welcoming space to share our thoughts, students were encouraged to reflect on our role in moving forward as anti-racist students and future physicians.

This program was incredible. I did not really know what to anticipate, but I learned more than I could have imagined. We learned a lot about mental health conditions, diagnoses, and medications, and in a more in-depth manner than we will in school. This program, however, went beyond that. It delved into WHY people are diagnosed--the signs and symptoms, but also due to biases, race, culture, and so many other factors that we don’t get a chance to explore in school.

I did not know much about psychiatry in youth. I honestly knew what people say about it--including medication and how it would be so hard to see kids struggling with mental health. I did not realize how much HOPE is in this field.

## Discussion

The availability of pediatric mental health care is a public health problem and national emergency. Youth and families deserve timely, quality, culturally responsive and respectful care. Early exposure programs may be one way to address the critical workforce shortage of CAPs with 70% of counties in the USA lacking a single CAP [4]. This immersive CAP summer program for medical students offered a new opportunity to learn about CAP and the importance of pediatric mental health. All respondents reported they would be more likely to pursue a career in CAP because of participating in this summer program. Many had not considered CAP or known it was a specialty to pursue prior to the call for applications.

Early enrichment programs appear to play a role in increasing or encouraging interest in psychiatry [17, 18]. Similar to other programs aimed at increasing exposure or interest in psychiatry, this early exposure opportunity was positively received. Emphasis on growing an inclusive and diverse workforce is essential to increase access and deliver equitable care.

Even if students exposed to youth and family mental health educational experiences during medical school do not eventually become CAPs, they may enter their respective specialties with an appreciation of the importance of youth and family well-being and may have greater comfort levels in discussing mental health, identifying concerns, and connecting patients to the appropriate services. Discussing barriers to choose CAP as a group was useful, including addressing professional and societal stigma, lack of exposure, and emotional challenges with the work. Cultivating inclusive clinical learning environments that include opportunities for reflection and processing may build empathy, increase resilience, and decrease stigma. Community cohesion was strong in this cohort; there was a clear sense of camaraderie, support, and genuine care for colleagues. Creating space to discuss one’s own identity and culture and how this relates to delivering sensitive, affirming, and empowering care may be worthwhile.

Further study is needed to explore the expansion and replicability of the program components and curriculum as well as any longitudinal impact on interest, increasing diversity, and trainees developing into more inclusive physician leaders. Limitations of this educational case report are numerous. This was a one-time experience without ability to generalize to other groups or schools. The cost of the speakers and stipends for the students would not have been possible without grant support from UnitedHealth Group. In addition, all students had some level of baseline interest in CAP given their application to the program.

In summary, choosing a medical specialty to pursue is a complex process that may begin before matriculation into medical school. Increasing opportunities to learn about CAP early in one’s medical journey may help grow the workforce and/or increase awareness about the importance of mental health and well-being across all medical specialities.

## References

[1] World Health Organization. Mental Health Atlas. 2017. [Accessed 1 June 2021]. Available from: https://www.who.int/publications/i/item/9789241514019.

[2] American Academy of Child and Adolescent Psychiatry. 2021. Workforce Maps by State. [Accessed on 30 May 2021]. Available from: https://www.aacap.org/aacap/Advocacy/Federal_and_State_Initiatives/Workforce_Maps/Home.aspx.

[3] Association of American Medical Colleges. Workforce: Physician Specialty Data Report. 2020. [Accessed on 8 June 2021]. Available from: https://www.aamc.org/what-we-do/mission-areas/health-care/workforce-studies/interactive-data/number-people-active-physician-specialty-2019.

[4] McBain RK, Kofner A, Stein BD, Cantor JH, Vogt WB, Yu H. Growth and distribution of child psychiatrists in the United States: 2007–2016. Pediatrics. 2019;144(6):e20191576. 10.1542/peds.2019-1576. 31685696 10.1542/peds.2019-1576 PMC6889947

[5] Axelson, D. Beyond a bigger workforce: addressing the shortage of child and adolescent psychiatrists. 2020. Pediatrics Nationwide. [Accessed 28 May 2021]. Available from: https://pediatricsnationwide.org/wp-content/uploads/2021/02/W154476_Pediatrics-Nationwide-Spring_Summer-2020-Single-Pages.pdf.

[6] Axelson D. Meeting the demand for pediatric mental health care. Pediatrics. 2019;144(6):e20192646. 31685697 10.1542/peds.2019-2646

[7] Health Resources & Services Administration. Behavioral health workforce projections, 2016–2030: psychiatrists (adult), child and adolescent psychiatrists. 2018. [Accessed 28 May 2021]. Available at: https://bhw.hrsa.gov/sites/default/files/bhw/nchwa/projections/psychiatrists-2018.pdf.

[8] Association of American Medical Colleges. Diversity in medicine: facts and figures. 2018. [Accessed 30 May 2021]. Available from: https://www.aamc.org/data-reports/workforce/interactive-data/figure-20-percentage-physicians-sex-and-race/ethnicity-2018.

[9] Wyse R, Hwang WT, Ahmed AA, Richards E, Deville C. Diversity by race, ethnicity, and sex within the US psychiatry physician workforce. Acad Psychiatry. 2020;44(5):523–30. 32705570 10.1007/s40596-020-01276-z

[10] Census [Internet]. 2021. [Accessed 15 May 2021]. Available from: https://www.census.gov/newsroom/press-releases/2021/population-changes-nations-diversity.html.

[11] Child Population, by Race/Ethnicity - Kidsdata.org [Internet]. 2020. [Accessed 15 May 2021]. Available from: https://www.kidsdata.org/topic/33/child-population-race/pie#fmt=144&loc=2&tf=110&ch=7,11,726,10,72,9,73,87&pdist=73.

[12] Jones N, Marks R, Ramirez R; Ríos-Vargas M. Unites States Census Bureau. 2020. Census Illuminates Racial and Ethnic Composition of the Country. [Accessed 14 May 2021]. Available from: https://www.census.gov/library/stories/2021/08/improved-race-ethnicity-measures-reveal-united-states-population-much-more-multiracial.html.

[13] Vespa, Jonathan, Armstrong, David M, Media L, United States Census Bureau. 2020. Demographic turning points for the United States: population projections for 2020 to 2060. [Accessed 15 May 2021]. Available from: https://www.census.gov/content/dam/Census/library/publications/2020/demo/p25-1144.pdf.

[14] Gomez LE, Bernet P. Diversity improves performance and outcomes. J Natl Med Assoc. 2019;111(4):383–92. 30765101 10.1016/j.jnma.2019.01.006

[15] Sawyer MG, Giesen F, Walter G. Child psychiatry curricula in undergraduate medical education. J Am Acad Child Adolesc Psychiatry. 2008;47(2):139–47. 18176334 10.1097/chi.0b013e31815cd9e0

[16] Volpe T, Boydell KM, Pignatiello A. Choosing child and adolescent psychiatry: factors influencing medical students. J Can Acad Child Adolesc Psychiatry. 2013;22(4):260–7. 24223044 PMC3825465

[17] Stein JA, Althoff R, Anders T, et al. Does early mentorship in child and adolescent psychiatry make a difference? The Klingenstein Third-Generation Foundation Medical Student Fellowship Program. Acad Psychiatry. 2013;37(5):321–4. 24026370 10.1176/appi.ap.12070136

[18] Lyons Z. Establishment and implementation of a psychiatry enrichment programme for medical students. Australas Psychiatry. 2017;25(1):69–72. 27679635 10.1177/1039856216671663

[19] Farooq K, Lydall GJ, Malik A, Ndetei DM, ISOSCCIP Group, Bhugra D. Why medical students choose psychiatry - a 20 country cross-sectional survey. BMC Med Educ. 2014;14:12. 24422951 10.1186/1472-6920-14-12 PMC3974144

[20] Lokko HN, Chen JA, Parekh RI, Stern TA. Racial and ethnic diversity in the US psychiatric workforce: a perspective and recommendations. Acad Psychiatry. 2016;40(6):898–904. 27421839 10.1007/s40596-016-0591-2

